# An Account of the Effects of Oil of Turpentine in a Case of Internal Hæmorrhage

**Published:** 1793

**Authors:** William Adair

**Affiliations:** Surgeon General to the Garrison of Gibraltar.


### III. *An Account of the Effects of Oil of Turpentine in a Case of internal Hæmorrhage. By the same.*

I have lately had an opportunity of proving the efficacy of oil of turpentine in a case of internal hæmorrhage. The patient, Mr. Delamar, of this place, is about forty-eight years of age. He was seized in March last, during a very cool, mild season, with sickness, vomiting, and pain in the lower part of the belly, followed by a discharge, by stool, of not less than a pound of black, coagulated blood, without any appearance of fæces. When I visited him, about three hours after the first stool, he had had two more stools resembling the first, except that the blood voided by the last stool was not coagulated, but frothy and high coloured. His extremities were cold; his pulse, at the wrist, was hardly perceptible; he had a hiccup, and seemed to be in a dying state.

I ordered ten drops of oil of turpentine to be given immediately; and as his stomach, since the attack, had rejected whatever he had taken, I directed the oil to be dissolved with a little of the yolk of an egg, and diluted with cinnamon water. In this form it was retained; and a surgeon of one of the regiments here, kindly undertook to sit up with him, and see the same dose repeated every six hours, or oftener, if the hæmorrhage should not abate.

When I visited the patient the next morning, I found that the discharge having diminished after the first dose, the medicine had been repeated only three times, and that there was hardly any blood in a stool he had voided that morning. His pulse was now full, but quick. The medicine was directed to be repeated only twice in twenty-four hours, and the next day it was entirely laid aside. But on that day (being the third from the attack) as he was costive, and had symptoms of fever, I thought it right to direct a gentle laxative medicine to open the bowels. This effect it produced, but it brought on bloody stools again with almost as much violence as at first. They were immediately stopped, however, by the oil of turpentine given in the same dose and form as before. After this I did not venture to prescribe for the patient any laxative medicine, by the mouth, for a considerable time; but contented myself with recommending the occafional ufe of clyfters, to obviate coftivenefs, till there feemed to be no longer any danger of the return of the hæmorrhage.

The patient is now perfectly recovered.

*Gibraltar,*
*November* 3, 1792.

